# Renal and Neurological Response with Eculizumab in a Patient with Transplant Associated Thrombotic Microangiopathy after Allogeneic Hematopoietic Progenitor Cell Transplantation

**DOI:** 10.1155/2015/425410

**Published:** 2015-01-29

**Authors:** Ömür Gökmen Sevindik, İnci Alacacıoğlu, Abdullah Katgı, Şerife Medeni Solmaz, Celal Acar, Özden Pişkin, Mehmet Ali Özcan, Fatih Demirkan, Bülent Ündar, Güner Hayri Özsan

**Affiliations:** Department of Hematology, Dokuz Eylül University, 35340 Izmir, Turkey

## Abstract

Transplantation-associated thrombotic microangiopathy (TA-TMA) is a challenge after allogeneic hematopoietic progenitor cell transplantation, considering the diagnostic uncertainties and lack of established treatment. We report a 43-year-old male patient who was diagnosed as TA-TMA after allogeneic progenitor cell transplantation for a progressive ALK negative anaplastic large cell lymphoma and responded to eculizumab with dramatically improving neurological status and renal function. Rapid neurological and renal recovery achieved after eculizumab could support a possible relationship between complement activation and TA-TMA. Eculizumab should be a reasonable treatment approach in patients with TA-TMA after allogeneic hematopoietic progenitor cell transplantation.

## 1. Introduction

Transplantation-associated thrombotic microangiopathy (TA-TMA) represents a challenge after allogeneic hematopoietic progenitor cell transplantation because of diagnostic uncertainties, lack of established treatment, and an overall poor prognosis [1]. Eculizumab, which is a monoclonal antibody against terminal complement, has been used successfully in atypical hemolytic and uremic syndrome and TA-TMA associated with solid organ transplantation. Data about its efficacy in TA-TMA associated with hematopoietic progenitor cell transplantation lacks [2].

## 2. Case Report

We report a 43-year-old male patient who was diagnosed as TA-TMA after allogeneic progenitor cell transplantation from HLA matched sibling donor and treated with eculizumab.

**Figure 1:**
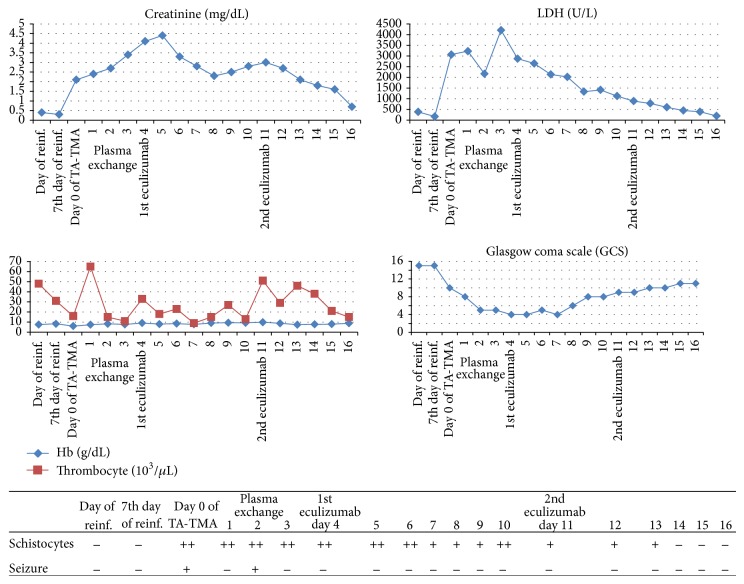
Clinical and biological response to eculizumab therapy. The figure shows data indicating neurological and renal improvement after administration of monoclonal C5 antibody eculizumab in our case with TA-TMA posthematopoietic progenitor cell transplantation with central nervous syprogenitor involvement. LDH levels were decreased dramatically, but thrombocyte count did not improve, possibly related to delayed hematopoietic engraftment.

The patient was diagnosed with stage 4 ALK negative anaplastic large cell lymphoma after splenectomy due to uncontrolled splenic hemorrhage. Considering the aggressive nature of the disease, CHOP regimen initiated immediately after diagnosis and staging procedures. After two cycles of CHOP regimen, no significant response was achieved and ESHAP regimen initiated. After two cycles of ESHAP regimen, a short term partial response could be achieved and allogeneic progenitor cell transplantation was applied from HLA matched (10/10) sibling donor of the patient because of progressive disease [3]. Cells were collected peripherally and the amount of reinfused CD34+ cells was 5.3 × 10^6^/kg. Busulfan and cyclophosphamide were used as conditioning and cyclosporine used for the prophylaxis of graft versus host disease. At the 7th day of reinfusion, acinetobacter sepsis developed and patient was put on to a broad spectrum of antibiotics. While recovering from acinetobacter sepsis, a rapid neurological and renal deterioration was observed. Patient was stuporous and had a tonic-clonic epileptic seizure; he also had a severe headache and hallucinations before the onset of seizure. Meanwhile, the ldh level was increased dramatically up to 3200 U/L and schistocytes were detected at the peripheral blood smear (20–30/HPF). Despite being hyponormotensive during the period of sepsis, he had hypertensive episodes with a maximum of 180/100 mmHg accompanying the neurological symptoms. Cranial MRI and lumbar puncture revealed no pathological feature. CSF culture was negative and cytological analysis revealed no malignant cells. Viral analysis of CSF and blood was negative regarding CMV and EBV. His Hb level has decreased from 9.5 to 7.2 g/dL with an MCV of 96 fL and reticulocyte count of 2.49%. Both direct and indirect coombs tests were negative and haptoglobulin level was slightly decreased below the lower limit of normal. After ruling out an ongoing infectious disease, cerebrovascular event, and malignant infiltration of CNS with lymphoma, patient was diagnosed, as TA-TMA. ADAMTS13 activity was %37 with no detectable inhibitors. All complement levels were in normal range (C3, C4, Factor H, and Factor I). Urinalysis revealed a protein concentration of 120 mg/dL. Calcineurin inhibitor stopped immediately after diagnosis and plasma exchange was initiated. Plasma exchange was performed on a daily basis with 1.5 plasma volumes, using FFP as a replacement fluid. Neurological status got worse and patient was intubated with a Glasgow coma scale (GCS) score of 4. Hemodialysis was applied due to worsening renal functions and acidosis. Despite rigorous plasma exchange, no response was achieved and plasma exchange was stopped after 5 consecutive cycles. Eculizumab therapy was initiated according to atypical hemolytic and uremic syndrome dosage. With two weekly administrations of 900 mgs of eculizumab, neurological status got gradually better and patient was able to achieve a GCS score of 11. Hemodialysis stopped and a normal glomerular filtration rate could be achieved. Despite recovering renal and neurological status, platelet count remained below 20000/microL, possibly related to delayed hematopoietic engraftment (Figure 1). Patient was lost with diffuse alveolar hemorrhage at the 12th day of eculizumab with a stable low platelet count, a normal ldh level, a normal glomerular filtration rate, a GCS score of 11, and fewer schistocytes at peripheral blood smear.

## 3. Discussion

Hematopoietic progenitor cell transplantation- (HPCT-) associated TA-TMA is a well-recognized and potentially severe complication of HPCT that can lead to a high risk of morbidity and mortality [4]. The incidence of TA-TMA is 10–35% as reported in the largest retrospective reviews and studies examining renal and other tissues [1]. Jodele et al. have reported a 39% incidence in 100 consecutive children and young adult patients who had hematopoietic progenitor cell transplantation [5]. The cause of TA-TMA is multifactorial. Risk factors include high-dose chemotherapy, radiation therapy, unrelated donor, HLA mismatch, exposure to calcineurin inhibitors (CNIs), graft-versus-host disease, and infections [1, 6–8]. The release of inflammatory cytokines causes microvascular endothelial injury and activates platelets and coagulation factors, leading to thrombosis and fibrin deposition in microvasculature of organs, most commonly in the kidney [1]. Recently, Jodele et al. and Laskin et al. have suggested an involvement of the complement dysregulation in the development of TA-TMA [9, 10]. Limited feasibility of tissue diagnosis of HPC-associated TA-TMA has led to the development of noninvasive diagnostic criteria. Cho et al. had validated the previously described criteria and added “probable TA-TMA” category, which did not require renal and neurological findings for the diagnosis of TA-TMA. Validated criteria included (1) lactate dehydrogenase (LDH) elevated above the upper limit of normal for age, (2) de novo thrombocytopenia with a platelet count <50 × 10^9^/L or a ≥50% decrease in the platelet count, (3) de novo anemia with a hemoglobin below the lower limit of normal or anemia requiring transfusion support, (4) microangiopathic changes defined as the presence of schistocytes in the peripheral blood or histologic evidence of microangiopathy on a tissue specimen, and (5) absence of coagulopathy and a negative Coombs test [6]. Plasma ADAMTS13 activity should be measured to rule out a possible diagnosis of TTP, even though TTP is very rare after HPCT [1, 11]. TA-TMA usually develops around day +60, but early (day +4) and late (2 years) episodes have been described. TA-TMA has been shown to be associated with a high mortality rate of up to 100% with a median of 75% [1, 4]. Over the last 30 years, several pharmacological and nonpharmacological treatment modalities for managing TA-TMA have been reported, but there is a paucity of data supporting an optimal treatment strategy. The most effective measure is to stop CNI immediately (no dose reduction) by changing GVHD prophylaxis/treatment to another drug (corticosteroids, mycophenolate). Plasma exchange usually offers a poor response (median: 35%; range: 20–80%) probably because TMA is not associated with an absence or severe reduction of plasma ADAMTS13 activity and has a high associated mortality (80%). Some authors have reported successful results with defibrotide, rituximab, daclizumab, and basiliximab therapies [12]. Mulay et al. have reported 24% clinical improvement rate with PE in 33 TA-TMA patients [13]. There is only one case defining the efficacy of eculizumab in an adult patient with postallogeneic progenitor cell transplantation which have developed thrombotic microangiopathy at the 3rd month of reinfusion. Within 48 hours of administering first dose of eculizumab, neurological symptoms and laboratory variables improved dramatically. His neurological status normalized after 2 weeks, and TMA completely resolved 3 months later [14]. In a case series of six pediatric HPCT recipients who developed TA-TMA, 67% complete remission were achieved with eculizumab, all of whom achieved complete blockade of complement activity [15]. To our knowledge, our case is the first in the literature defining the efficacy of eculizumab at thrombotic microangiopathy which developed rapidly after reinfusion. Rapid neurological and renal recovery achieved after eculizumab could support a possible relationship between complement activation and TA-TMA. Eculizumab should be a reasonable treatment approach in patients with TA-TMA after allogeneic hematopoietic progenitor cell transplantation.
